# Altered Serum Phospholipids in Atopic Dermatitis and Association with Clinical Status

**DOI:** 10.1016/j.xjidi.2021.100092

**Published:** 2021-12-22

**Authors:** Takashi Sakai, Nadine Herrmann, Laura Maintz, Tim Joachim Nümm, Thomas Welchowski, Ralf A. Claus, Markus H. Gräler, Thomas Bieber

**Affiliations:** 1 Department of Dermatology and Allergy, Christine Kühne-Center for Allergy Research and Education (CK-CARE), University Hospital Bonn, Bonn, Germany; 2 Department of Dermatology, Faculty of Medicine, Oita University, Oita, Japan; 3 Department of Medical Biometry, Informatics and Epidemiology, University Hospital Bonn, Bonn, Germany; 4 Department of Anesthesiology and Intensive Care Medicine, Center for Sepsis Control and Care (CSCC), and the Center for Molecular Biomedicine (CMB), Jena University Hospital, Jena, Germany

**Keywords:** AD, atopic dermatitis, Cer, ceramide, EASI, eczema area and severity index, LPC, lysophosphatidylcholine, LPE, lysophosphatidylethanolamine, PC, phosphatidylcholine, S1P, sphingosine-1-phosphate, Sph, sphingosine

## Abstract

Circulating phospholipids have been considered as biomarkers and therapeutic targets in multiple disorders. Atopic dermatitis (AD) is the most common inflammatory skin disease. Although there are numerous studies having addressed stratum corneum lipids in the context of epidermal barrier, little is known about the circulating lipids in patients with AD. In this study, we explored the changes of serum phospholipids in AD using liquid chromatography coupled to tandem mass spectrometry and sought serum lipids’ contribution to clinical status. Several serum levels of phospholipids were altered in the AD group (n = 179) compared with that in healthy controls (n = 47) and patients without AD with atopic comorbidities (n = 22); lipids exhibiting the apparent changes included increased sphingosine, multiple variants of phosphatidylcholine, and decreased ceramide (16:0) in patients with AD. Moreover, serum levels of sphingosine correlated with the severity of AD, and sphingosine and ceramide(16:0) were also detected as the risk-increasing effect and risk-reduction effect of AD, respectively. In summary, alterations in the serum concentration of phospholipids are seen in patients with AD. Although more detailed investigations will be needed to evaluate the significance of the changes in circulating lipids in AD, these findings can provide, to our knowledge, previously unreported insight into AD’s pathogenesis and therapeutic strategies.

## Graphical abstract

**Figure:**
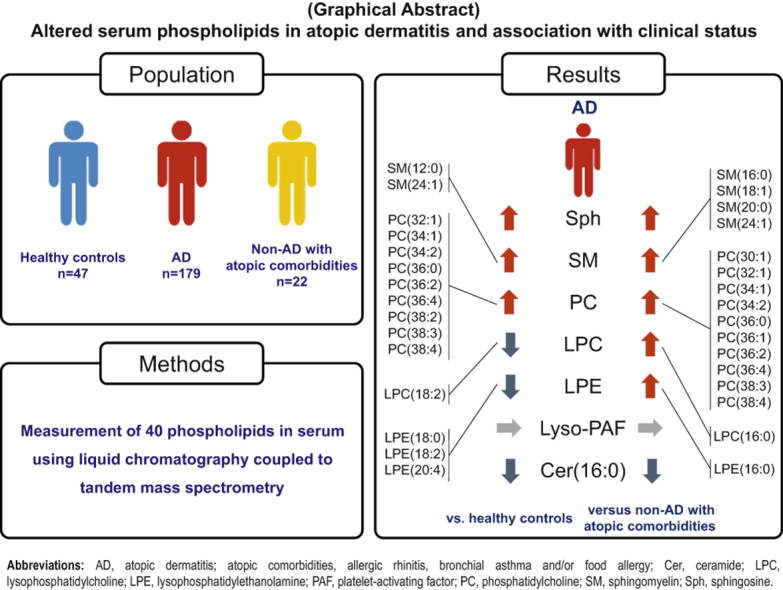

## Introduction

Atopic dermatitis (AD) is the most common inflammatory skin disease with a strong impact on the QOL of patients and their relatives and a significant socioeconomic burden. Epidermal barrier dysfunction and T helper 2‒mediated inflammation-inducing pruritus are hallmarks of AD. In addition, AD is often associated with comorbidities such as allergic rhinitis, bronchial asthma, or food allergy and subsequent physical and psychological pain (Weidinger et al., 2018). Although there are numerous studies having addressed stratum corneum lipids in the context of epidermal barrier (Borodzicz et al., 2016; Park et al., 2019), little is known about the circulating lipids in patients with AD. Metabolomics is an approach for studying metabolic changes connected to clinical significance as well as for finding biomarkers, and it can provide new insight into diseases’ pathogenesis. It is well-known that circulating levels of ceramide (Cer) and sphingolipids have emerged as predictive and diagnostic biomarkers of cardiometabolic complications, including coronary artery disease, diabetes, and heart failure (Choi et al., 2021) and several neurodegenerative disorders, including Alzheimer's disease and Parkinson's disease (Alessenko and Albi, 2020). Those sphingolipids in cardiovascular and neurodegenerative disorders have also been considered therapeutic targets (Alessenko and Albi, 2020; Choi et al., 2021). Furthermore, disturbances in circulating sphingolipids levels and their association with the phenotype have been observed in inflammatory and allergic disorders, such as systemic lupus erythematosus (Watson et al., 2012), psoriasis (Kowalski et al., 2013; Myśliwiec et al., 2017), or bronchial asthma (Kim et al., 2020; Kowal et al., 2019). Recently, we reported that serum concentrations of sphingosine (Sph)-1-phosphate (S1P), which is a bioactive lipid mediator, in patients with AD are higher than those in healthy individuals and patients without AD with atopic comorbidities (Sakai et al., 2021). S1P receptors are therapeutic targets in multiple disorders (Cartier and Hla, 2019), and the clinical trials with the compounds targeting S1P receptors in patients with AD are underway (NCT04162769, ACTRN12617000763347). Although other serum phospholipid levels have been evaluated in animal models with allergic dermatitis—eczema horses had significantly lower concentrations of phosphatidylcholine (PC) and sphingomyelin than their healthy controls (Hallamaa and Batchu, 2016)—they have not been examined in patients with AD. We hypothesized that serum phospholipids in patients with AD are altered, which may be associated with clinical findings of AD. In this study, we explored the changes of serum phospholipids in AD with metabolomics and sought serum lipids’ contribution to clinical status.

## Results

### Altered serum levels of phospholipids in patients with AD

**Figure 1:**
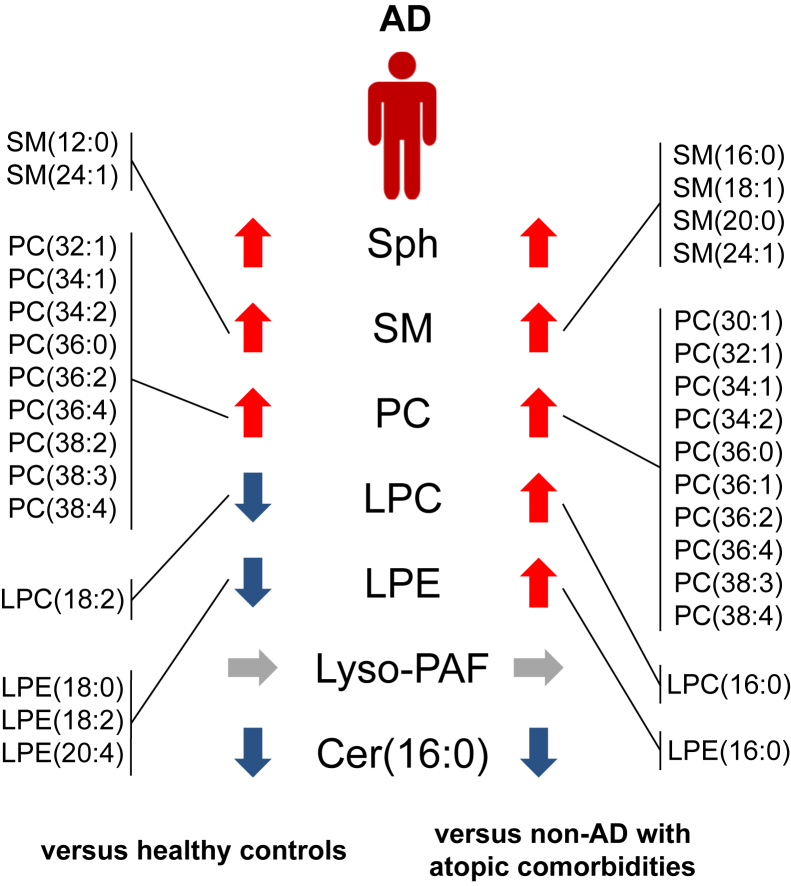
**Several serum lipids concentrations are altered in patients with AD.** We analyzed 40 lipids concentrations in serum from healthy individuals, individuals with AD, and individuals with non-AD with atopic comorbidities (allergic rhinitis, bronchial asthma, and/or food allergy). This figure is a graphical summary of altered lipids in the sera of the AD group compared with those of the healthy controls or non-AD with atopic comorbidities group, summarizing Table 1. AD, atopic dermatitis; Cer, ceramide; LPC, lysophosphatidylcholine; LPE, lysophosphatidylethanolamine; PAF, platelet-activating factor; PC, phosphatidylcholine; SM, sphingomyelin; Sph, sphingosine.

This study consisted of three groups: healthy controls (n = 47), those with AD (n = 179), and those with non-AD with atopic comorbidities (i.e., non-AD with allergic rhinitis, bronchial asthma, and/or food allergy, n = 22). We recruited these participants as a part of the Christine Kühne-Center for Allergy Research and Education (Davos, Switzerland) program (Bieber et al., 2020), who were the same as those recruited in our previous study (Sakai et al., 2021). We evaluated the serum concentrations of 40 lipids in all participants (Table 1). Patients with AD showed increased serum levels of Sph and multiple variants of sphingomyelin and PC and decreased serum levels of Cer(16:0), with statistical significance, compared with healthy controls and those with non-AD with atopic comorbidities. Serum levels of lyso-platelet-activating factor in the AD group were not changed compared with those in the healthy controls and non-AD with atopic comorbidities group. Regarding serum concentrations of lysophosphatidylcholine (LPC), one variant of LPC (LPC[18:2]) was decreased in AD group compared with that in healthy controls, whereas one variant of LPC (LPC[16:0]) was increased in AD group compared with that in non-AD with atopic comorbidities group. The serum levels of three variants of lysophosphatidylethanolamine (LPE) were decreased in the AD group compared with that in healthy controls, whereas one variant of LPE was increased in the AD group compared with that in the non-AD with atopic comorbidities group. About comparing between healthy controls and non-AD with comorbidities group, serum levels of four variants of LPE, lyso-platelet-activating factor, and Cer(16:0) were decreased in non-AD with atopic comorbidities group compared with that in healthy controls (Table 1 and Figure 1).

**Table 1:** Serum Levels of 40 Lipids in Healthy Controls, AD Group, and non-AD with Atopic Comorbidities Group

	Healthy Controls (n = 47, A) Median (Q1–Q3) (nM)	AD (n = 179, B) Median (Q1–Q3) (nM)	Non-AD with Atopic Comorbidities (n = 22, C) Median (Q1–Q3) (nM)	*P*-Value^1^ (A vs. B)	*P*-Value^1^ (B vs.C)	*P*-Value^1^ (A vs.C)
Sph	18.62 (15.73–22.54)	21.83 (17.44–26.65)	18.17 (14.21–21.33)	0.016	0.025	0.58
SM(12:0)	1,576 (967.5–1,913)	1,715 (1,264–2,405)	1,379 (882.8–1990)	0.038	0.065	0.74
SM(14:0)	38,238 (28,345–44,903)	34,913 (25,884–43,846)	29,018 (20,482–36,636)	0.57	0.059	0.029
SM(16:0)	51,429 (45,351–56,906)	49,451 (42,758–57,178)	41,448 (33,619–47,383)	0.49	0.001	<0.001
SM(18:1)	3,571 (3,132–3,969)	3663 (3,039–4,544)	3,141 (2,402–4,152)	0.39	0.037	0.18
SM(18:0)	6,405 (5,512–7,631)	6,066 (5,160–7,216)	5,464 (3,974–6,749)	0.14	0.19	0.054
SM(20:0)	11,209 (8,846–12,773)	11,498 (9,545–14,037)	8,616 (6,666–10,829)	0.39	0.002	0.020
SM(22:0)	8,810 (6,779–11,553)	9,131 (7,192–11,307)	8,992 (6,860–10,471)	0.93	0.62	0.75
SM(24:1)	18,905 (15,611–22,571)	20,433 (17,026–26,503)	17,209 (13,022–20,766)	0.038	0.008	0.15
SM(24:0)	4,199 (2,978–6,106)	4,367 (3,182–6,376)	4,858 (3,533–6,345)	0.91	0.66	0.77
PC(28:0)	1,446 (913.6–1,779)	1,579 (1,076–2,425)	1,229 (799.7–1,889)	0.26	0.087	0.49
PC(30:0)	2,240 (1,805–2,613)	2,440 (1928–2,948)	2,082 (1,525–2,746)	0.061	0.090	0.72
PC(30:1)	1,345 (917.6–1,832)	1,568 (1,105–2,219)	1,037 (822.8–1,694)	0.065	0.033	0.43
PC(32:0)	3,012 (2,753–3,197)	3,068 (2,690–3,460)	3,008 (2,491–3,502)	0.62	0.55	0.81
PC(32:1)	4,079 (2,881–5,294)	5,447 (4,192–8,760)	4,460 (2,664–5,656)	<0.001	0.017	0.95
PC(34:1)	60,114 (52,020–68,063)	72,821 (61,644–82,759)	56,169 (42,461–71,825)	<0.001	0.004	0.58
PC(34:2)	109,599 (100,154–124,717)	130,849 (111,398–153,846)	110,896 (92,448–139,205)	<0.001	0.032	0.86
PC(36:0)	1,440 (1,228–1,696)	1,801 (1,369–2,227)	1,265 (851.5–1,761)	0.002	0.006	0.23
PC(36:1)	5,669 (5,068–6,133)	5,913 (5,114–7,034)	4,894 (3,894–6,237)	0.079	0.030	0.19
PC(36:2)	3,071 (2,750–3,583)	3,630 (3,096–4,444)	2,965 (2,378–3,861)	<0.001	0.026	0.77
PC(36:4)	56,379 (48,538–66,667)	70,488 (58,537–84,896)	60,260 (44,664–73,690)	<0.001	0.020	0.60
PC(38:1)	2,522 (1,701–3,318)	2,296 (1,718–3,126)	2,897 (2,129–3,627)	0.66	0.11	0.33
PC(38:2)	10,343 (9,459–12,178)	11,679 (10,253–13,884)	10,517 (8,308–13,209)	0.009	0.091	0.94
PC(38:3)	6,768 (6,345–8,018)	8,205 (6,609–9,735)	6,326 (4,701–7,685)	0.011	0.002	0.097
PC(38:4)	22,953 (19,117–27,907)	29,309 (23,333–35,094)	23,072 (18,434–30,083)	<0.001	0.032	0.90
LPC(16:0)	13,321 (11,626–14,357)	13,235 (11,709–15,293)	12,077 (10,216–13,272)	0.53	0.032	0.091
LPC(16:1)	473 (379.5–574.3)	488.1 (381.5–620.1)	477.5 (283.5–531.1)	0.52	0.23	0.60
LPC(18:0)	7,324 (5,905–8,171)	6,881 (5,190–8,074)	5,458 (3,960–6,969)	0.37	0.087	0.033
LPC(18:1)	4,349 (3,455–4,921)	3,965 (3,280–4,979)	3,704 (2,656–4,417)	0.49	0.27	0.12
LPC(18:2)	6667 (5637–7,658)	5,767 (4,447–7,195)	5,810 (4,851–7,643)	0.011	0.91	0.14
LPC(20:1)	75 (61.98–91.64)	81.96 (61.68–97.21)	75.51 (53.76–106.5)	0.39	0.81	0.91
LPC(20:3)	514.9 (400.2–596.3)	482.1 (374.1–572.2)	448.1 (353.2–612.4)	0.16	0.84	0.39
LPC(20:4)	1,386 (1,068–1,589)	1209 (975.4–1,473)	1,439 (802.1–1,673)	0.079	0.57	0.84
LPE(16:0)	111.9 (96.21–135.4)	109.6 (88.05–133.5)	88.92 (60.88–106.9)	0.52	0.017	0.009
LPE(18:0)	46.48 (37.24–60.32)	39.04 (28.18–50.4)	30.7 (21.53–43.37)	0.011	0.10	0.006
LPE(18:1)	143.7 (89.15–171.7)	123.3 (90.59–154.1)	109.8 (80.64–143.1)	0.27	0.43	0.17
LPE(18:2)	256.8 (206.5–329.5)	209.5 (157.8–281.2)	189.4 (145.3–246.7)	0.010	0.59	0.048
LPE(20:4)	136.7 (106.6–168.9)	113.5 (88.27–144.2)	104.5 (87.9–141)	0.009	0.55	0.014
Lyso-PAF	16.54 (13.97–21.46)	16.82 (12.5–21.39)	13.91 (10.41–17.07)	0.49	0.079	0.033
Cer(16:0)	302.5 (250.4–348)	161.9 (104–220.8)	237.7 (202.7–277.9)	<0.001	<0.001	0.006

Abbreviations: AD, atopic dermatitis; Cer, ceramide; LPC, lysophosphatidylcholine; LPE, lysophosphatidylethanolamine; PAF, platelet-activating factor; PC, phosphatidylcholine; SM, sphingomyelin; Sph, sphingosine.

1 Significances were calculated by the Kruskal‒Wallis test followed by pairwise comparisons with Mann‒Whitney U test. The *P*-values were adjusted using Benjamini‒Hochberg procedure.

### Correlation between serum phospholipid levels and clinical status in AD

**Figure 2:**
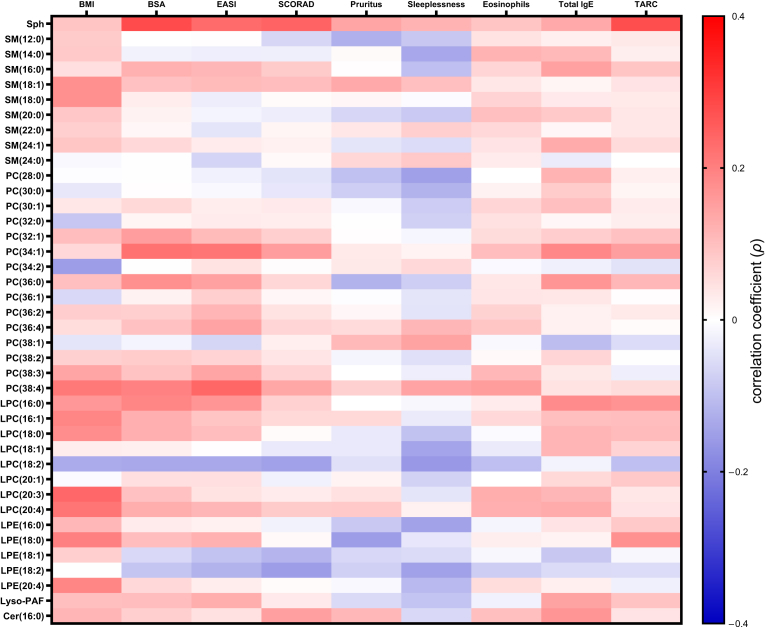
**Correlation between serum lipids concentrations and clinical and laboratory data in patients with AD.** The heatmap shows the correlation between serum lipids concentrations and clinical/laboratory data in patients with AD. Color red to blue indicates correlation coefficient (*ρ*). Correlations were analyzed by Spearman’s correlation test. The *P*-values for multiple comparisons were adjusted using Benjamini‒Hochberg procedure. The *ρ* values and *P*-values are shown in Tables 2 and 3. AD, atopic dermatitis; BMI, body mass index; BSA, body surface area; Cer, ceramide; EASI, eczema area and severity index; LPC, lysophosphatidylcholine; LPE, lysophosphatidylethanolamine; PAF, platelet-activating factor; PC, phosphatidylcholine; SCORAD, severity scoring of atopic dermatitis; SM, sphingomyelin; Sph, sphingosine; TARC, thymus and activation-regulated chemokine.

Next, we evaluated the correlation between these serum lipids concentrations and clinical and laboratory data in AD (Figure 2 and Tables 2 and 3). Body mass index showed no correlation with all evaluated serum phospholipid levels, with statistical significance. Although correlation coefficients were low, serum levels of Sph positively correlated with body surface area (*ρ* = 0.281, *P* = 0.032) and serum levels of thymus and activation-regulated chemokine (*ρ* = 0.277, *P* = 0.032). Other serum phospholipid levels showed no significant correlation with any clinical and laboratory data (Figure 2 and Tables 2 and 3).

**Table 2:** Correlation between Serum Lipids Concentrations and Clinical and Laboratory Data in Patients with AD (*ρ* Values)

	BMI	BSA	EASI	SCORAD	Pruritus	Sleeplessness	Eosinophils	Total IgE	TARC
Sph	0.092	0.281	0.231	0.242	0.140	0.117	0.092	0.132	0.277
SM(12:0)	0.082	–0.001	0.001	–0.064	–0.125	–0.089	0.045	0.025	0.035
SM(14:0)	0.085	–0.021	–0.027	–0.026	0.007	–0.139	0.119	0.109	0.026
SM(16:0)	0.049	0.123	0.114	0.083	0.002	–0.101	0.065	0.146	0.090
SM(18:1)	0.173	0.096	0.108	0.102	0.133	0.101	0.037	0.015	0.041
SM(18:0)	0.174	0.027	–0.029	0.006	0.013	–0.006	0.068	0.032	0.033
SM(20:0)	0.087	0.019	–0.018	–0.029	–0.065	–0.086	0.101	0.084	0.039
SM(22:0)	0.075	0.011	–0.042	0.015	0.037	0.074	0.058	0.013	0.037
SM(24:1)	0.089	0.057	0.030	0.022	–0.042	–0.056	0.041	0.131	0.055
SM(24:0)	–0.011	–0.002	–0.069	0.009	0.062	0.085	0.031	–0.032	0.000
PC(28:0)	–0.004	–0.002	–0.022	–0.043	–0.100	–0.150	0.000	0.117	0.025
PC(30:0)	–0.039	0.001	–0.012	–0.038	–0.078	–0.122	0.020	0.081	0.015
PC(30:1)	0.037	0.061	0.028	0.032	–0.011	–0.081	0.063	0.101	0.030
PC(32:0)	–0.090	0.015	0.031	0.028	–0.002	–0.074	0.047	0.013	0.026
PC(32:1)	0.103	0.153	0.108	0.075	0.003	–0.015	0.054	0.077	0.095
PC(34:1)	0.057	0.221	0.218	0.153	0.034	0.018	0.102	0.187	0.151
PC(34:2)	–0.154	–0.003	0.043	0.004	0.036	0.058	–0.010	–0.025	–0.046
PC(36:0)	0.101	0.175	0.147	0.062	–0.122	–0.078	0.038	0.165	0.112
PC(36:1)	–0.060	0.019	0.076	0.012	–0.004	–0.042	0.040	0.039	0.001
PC(36:2)	0.077	0.075	0.115	0.045	0.011	–0.044	0.070	0.023	0.033
PC(36:4)	0.052	0.095	0.143	0.065	0.055	0.114	0.088	0.022	0.006
PC(38:1)	–0.042	–0.018	–0.066	0.024	0.109	0.145	–0.012	–0.104	–0.056
PC(38:2)	0.073	0.079	0.069	0.040	–0.017	–0.049	0.008	0.063	–0.002
PC(38:3)	0.141	0.094	0.140	0.069	0.000	–0.028	0.110	0.036	–0.027
PC(38:4)	0.209	0.196	0.240	0.139	0.073	0.142	0.154	0.043	0.054
LPC(16:0)	0.163	0.190	0.166	0.072	0.000	–0.014	0.031	0.180	0.168
LPC(16:1)	0.191	0.125	0.090	0.061	0.061	–0.034	0.057	0.099	0.103
LPC(18:0)	0.178	0.125	0.102	0.005	–0.036	–0.096	–0.007	0.116	0.106
LPC(18:1)	0.028	0.025	0.001	–0.035	–0.035	–0.143	–0.034	0.115	0.068
LPC(18:2)	–0.133	–0.138	–0.137	–0.148	–0.049	–0.162	–0.101	–0.020	–0.101
LPC(20:1)	–0.009	0.048	0.050	–0.022	0.016	–0.071	–0.001	0.061	0.086
LPC(20:3)	0.236	0.095	0.043	0.031	0.046	–0.041	0.127	0.114	0.037
LPC(20:4)	0.215	0.128	0.113	0.082	0.084	0.023	0.124	0.131	0.042
LPE(16:0)	0.111	0.031	0.022	–0.021	–0.087	–0.147	–0.016	0.042	0.085
LPE(18:0)	0.199	0.102	0.122	0.008	–0.155	–0.038	0.026	0.017	0.172
LPE(18:1)	0.076	–0.060	–0.095	–0.117	–0.063	–0.057	–0.014	–0.089	–0.013
LPE(18:2)	–0.002	–0.091	–0.122	–0.154	–0.074	–0.148	–0.079	–0.057	–0.055
LPE(20:4)	0.190	0.059	0.027	0.005	–0.011	–0.110	0.052	0.024	–0.024
Lyso-PAF	0.099	0.103	0.126	0.032	–0.059	–0.094	–0.022	0.145	0.093
Cer(16:0)	0.115	0.074	0.046	0.150	0.110	–0.061	0.100	0.164	0.041

Abbreviations: AD, atopic dermatitis; BMI, body mass index; BSA, body surface area; Cer, ceramide; EASI, eczema area and severity index; Ig, immunoglobulin; LPC, lysophosphatidylcholine; LPE, lysophosphatidylethanolamine; PAF, platelet-activating factor; PC, phosphatidylcholine; SCORAD, severity scoring of atopic dermatitis; SM, sphingomyelin; Sph, sphingosine; TARC, thymus and activation regulated chemokine.

We analyzed the correlation between serum lipids concentrations and clinical and laboratory data in patients with AD (n = 179). The *ρ* values were calculated by Spearman's correlation test, and the *P*-values were adjusted using Benjamini‒Hochberg procedure. Table 2 shows the *ρ* values supporting Figure 2.

**Table 3:** Correlation between Serum Lipids Concentrations and Clinical and Laboratory Data in Patients with AD (*P-*Values)

	BMI	BSA	EASI	SCORAD	Pruritus	Sleeplessness	Eosinophils	Total IgE	TARC
Sph	0.65	0.032	0.11	0.11	0.44	0.57	0.65	0.50	0.032
SM(12:0)	0.70	1.00	1.00	0.81	0.52	0.65	0.92	0.94	0.93
SM(14:0)	0.68	0.95	0.94	0.94	1.00	0.44	0.56	0.59	0.94
SM(16:0)	0.89	0.52	0.57	0.69	1.00	0.60	0.81	0.43	0.65
SM(18:1)	0.35	0.63	0.59	0.60	0.49	0.60	0.92	0.97	0.92
SM(18:0)	0.35	0.94	0.94	1.00	0.97	1.00	0.78	0.93	0.93
SM(20:0)	0.66	0.96	0.96	0.94	0.81	0.67	0.60	0.68	0.92
SM(22:0)	0.73	0.98	0.92	0.97	0.92	0.74	0.83	0.97	0.92
SM(24:1)	0.65	0.83	0.93	0.94	0.92	0.83	0.92	0.50	0.83
SM(24:0)	0.98	1.00	0.78	0.99	0.81	0.68	0.93	0.93	1.00
PC(28:0)	1.00	1.00	0.94	0.92	0.60	0.43	1.00	0.57	0.94
PC(30:0)	0.92	1.00	0.98	0.92	0.72	0.52	0.96	0.70	0.97
PC(30:1)	0.92	0.81	0.94	0.93	0.98	0.70	0.81	0.60	0.93
PC(32:0)	0.65	0.97	0.93	0.94	1.00	0.74	0.91	0.97	0.94
PC(32:1)	0.60	0.43	0.59	0.73	1.00	0.97	0.85	0.73	0.63
PC(34:1)	0.83	0.15	0.15	0.43	0.93	0.96	0.60	0.28	0.43
PC(34:2)	0.43	1.00	0.92	1.00	0.93	0.83	0.98	0.94	0.91
PC(36:0)	0.60	0.35	0.43	0.81	0.52	0.72	0.92	0.39	0.57
PC(36:1)	0.82	0.96	0.73	0.98	1.00	0.92	0.92	0.92	1.00
PC(36:2)	0.73	0.73	0.57	0.92	0.98	0.92	0.76	0.94	0.93
PC(36:4)	0.86	0.63	0.44	0.81	0.83	0.57	0.66	0.94	1.00
PC(38:1)	0.92	0.96	0.79	0.94	0.59	0.43	0.98	0.60	0.83
PC(38:2)	0.74	0.72	0.78	0.92	0.97	0.89	1.00	0.81	1.00
PC(38:3)	0.44	0.63	0.44	0.78	1.00	0.94	0.59	0.92	0.94
PC(38:4)	0.18	0.25	0.11	0.44	0.74	0.44	0.43	0.92	0.85
LPC(16:0)	0.39	0.26	0.39	0.74	1.00	0.97	0.93	0.34	0.39
LPC(16:1)	0.26	0.52	0.65	0.81	0.81	0.93	0.83	0.61	0.60
LPC(18:0)	0.34	0.52	0.60	1.00	0.93	0.63	1.00	0.57	0.60
LPC(18:1)	0.94	0.94	1.00	0.93	0.93	0.44	0.93	0.57	0.78
LPC(18:2)	0.49	0.45	0.45	0.43	0.89	0.39	0.60	0.96	0.60
LPC(20:1)	0.99	0.90	0.88	0.94	0.97	0.76	1.00	0.81	0.68
LPC(20:3)	0.11	0.63	0.92	0.93	0.91	0.92	0.52	0.57	0.92
LPC(20:4)	0.15	0.52	0.57	0.70	0.68	0.94	0.52	0.50	0.92
LPE(16:0)	0.59	0.93	0.94	0.94	0.66	0.43	0.97	0.92	0.68
LPE(18:0)	0.25	0.60	0.52	0.99	0.43	0.92	0.94	0.97	0.35
LPE(18:1)	0.73	0.82	0.63	0.57	0.81	0.83	0.97	0.65	0.97
LPE(18:2)	1.00	0.65	0.52	0.43	0.74	0.43	0.72	0.83	0.83
LPE(20:4)	0.26	0.82	0.94	1.00	0.98	0.59	0.86	0.94	0.94
Lyso-PAF	0.61	0.60	0.52	0.93	0.82	0.63	0.94	0.43	0.64
Cer(16:0)	0.57	0.74	0.91	0.43	0.59	0.81	0.60	0.39	0.92

Abbreviations: AD, atopic dermatitis; BMI, body mass index; BSA, body surface area; Cer, ceramide; EASI, eczema area and severity index; Ig, immunoglobulin; LPC, lysophosphatidylcholine; LPE, lysophosphatidylethanolamine; PAF, platelet-activating factor; PC, phosphatidylcholine; SCORAD, severity scoring of atopic dermatitis; SM, sphingomyelin; Sph, sphingosine; TARC, thymus and activation regulated chemokine.

We analyzed the correlation between serum lipids concentrations and clinical and laboratory data in patients with AD (n = 179). The *ρ* values were calculated by Spearman's correlation test, and the *P*-values were adjusted using Benjamini‒Hochberg procedure. Table 3 shows the *P*-values supporting Figure 2.

### Serum levels of Sph is a risk-increasing effect of AD

**Figure 3:**
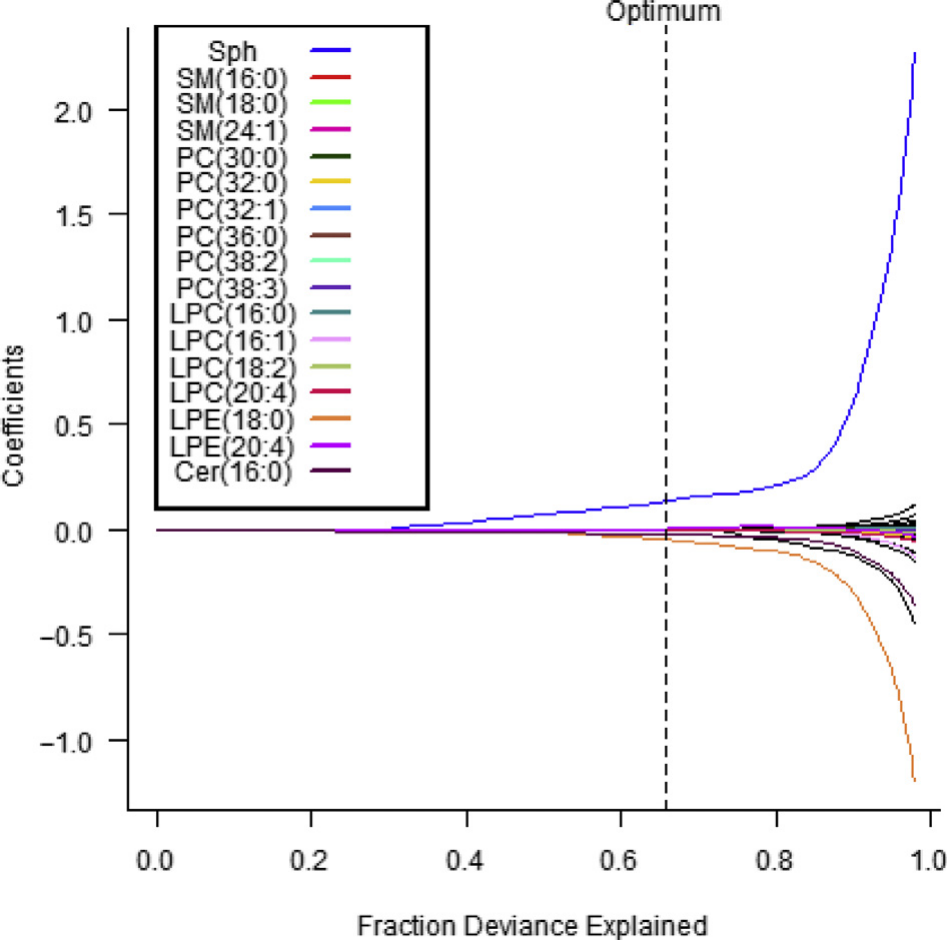
**Supporting figure for penalized Lasso regression.** This figure, supporting penalized Lasso regression (Table 4), shows how the coefficient paths depend on the data-dependent regularization parameter. The actual model estimates (optimum) of all coefficient paths that were nonzero (17 covariates) at the optimal solution were colored with the legend. Without Lasso penalty, the parameters would be estimated as the points at the end of each curve on the far right. Cer, ceramide; LPC, lysophosphatidylcholine; LPE, lysophosphatidylethanolamine; PC, phosphatidylcholine; SM, sphingomyelin; Sph, sphingosine.

**Figure 4:**
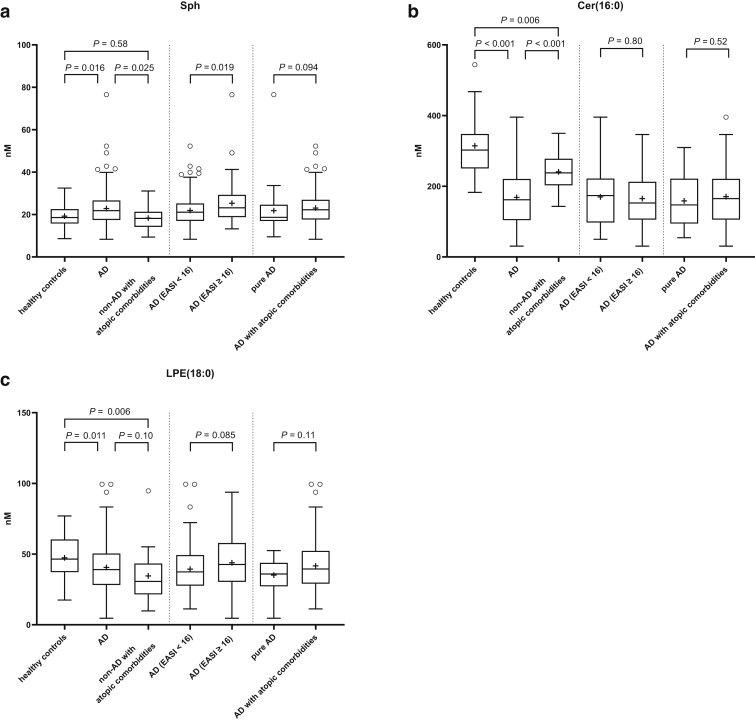
**Serum levels of Sph are elevated in moderate-to-severe AD.** We analyzed the serum concentrations of (**a**) Sph, (**b**) Cer(16:0), and (**c**) LPE(18:0) in the study groups: healthy individuals, patients with AD, and patients without AD with atopic comorbidities (allergic rhinitis, bronchial asthma and/or food allergy) or in the AD subgroups stratified by EASI or in the AD subgroups with or without atopic comorbidities. Significances were calculated with the two-tailed Mann‒Whitney U test (middle and right panels) or with the Kruskal‒Wallis test followed by pairwise comparisons with Mann‒Whitney U test. The *P*-values for multiple comparisons were adjusted using Benjamini‒Hochberg procedure (left panels). Healthy controls (n = 47), patients with AD (n = 179), patients with non-AD with atopic comorbidities (n = 22); AD (EASI < 16) (n = 130), AD (EASI ≥ 16) (n = 49); pure AD (n = 30), AD with atopic comorbidities (n = 149). AD, atopic dermatitis; Cer, ceramide; EASI, eczema area and severity index; LPE, lysophosphatidylethanolamine; Sph, sphingosine.

To evaluate which phospholipids have a strong association when predicting AD or non-AD (healthy controls and non-AD with atopic comorbidities group), we performed penalized Lasso regression. A total of 17 of 40 variables were selected that are associated with the risk of AD. The strongest risk-increasing effect was Sph, and the strongest risk-reduction effects were Cer(16:0) and LPE(18:0) (Table 4 and Figure 3). We also evaluated these serum lipids levels in the AD subgroups stratified by AD’s severity (AD [eczema area and severity index [EASI] < 16] vs. AD [EASI ≥ 16]) or atopic comorbidities (pure AD vs. AD with atopic comorbidities). Serum Sph levels were elevated in moderate to severe AD compared with those in mild to moderate AD. There were no differences in serum Cer(16:0) and LPE(18:0) levels between those AD subgroups (Figure 4).

**Table 4:** The Serum Phospholipids Being Associated with the Risk of AD

Variable	OR AD
Sph	1.142343555
LPE(20:4)	1.002415184
LPC(16:1)	1.001474071
PC(36:0)	1.000258849
PC(30:0)	1.000220845
LPC(16:0)	1.000218341
PC(38:3)	1.000179719
SM(24:1)	1.000111326
SM(16:0)	1.000056987
PC(32:1)	1.000043308
PC(38:2)	1.000011827
SM(12:0)	1
SM(14:0)	1
SM(18:1)	1
SM(20:0)	1
SM(22:0)	1
SM(24:0)	1
PC(28:0)	1
PC(30:1)	1
PC(34:1)	1
PC(34:2)	1
PC(36:1)	1
PC(36:2)	1
PC(36:4)	1
PC(38:1)	1
PC(38:4)	1
LPC(18:0)	1
LPC(18:1)	1
LPC(20:1)	1
LPC(20:3)	1
LPE(16:0)	1
LPE(18:1)	1
LPE(18:2)	1
Lyso-PAF	1
LPC(18:2)	0.999835522
SM(18:0)	0.999788959
PC(32:0)	0.99943743
LPC(20:4)	0.998199605
Cer(16:0)	0.976745322
LPE(18:0)	0.954168832

Abbreviations: AD, atopic dermatitis; Cer, ceramide; LPC, lysophosphatidylcholine; LPE, lysophosphatidylethanolamine; PAF, platelet-activating factor; PC, phosphatidylcholine; SM, sphingomyelin; Sph, sphingosine.

We sought the serum phospholipids being associated with the risk of AD with L1 penalized logistic regression between AD and non-AD, which includes healthy controls and those with non-AD with atopic comorbidities.

## Discussion

Several serum levels of phospholipids were altered in the AD group compared with those in the other groups; lipids exhibiting the apparent changes included increased Sph and multiple variants of PC and decreased Cer(16:0) in patients with AD. Moreover, serum levels of Sph, correlating with body surface area and thymus and activation-regulated chemokine, were elevated in moderate to severe AD, such as circulating S1P in AD (Sakai et al., 2021). Sph and Cer(16:0) were also detected as the risk-increasing effect and risk-reduction effect of AD, respectively. These findings suggest that altered serum levels of phospholipids might be involved in AD’s pathogenesis or biomarkers reflecting systemic inflammation owing to dermatitis.

The important roles of sphingolipids metabolites, especially in immune systems (Cinque et al., 2003) and as biomarkers in multiple disorders, have been well-established. Serum levels of Sph are associated with the symptomatic phenotypes of patients with COVID-19 (Janneh et al., 2021) and are fluctuated in patients with hepatitis B virus or hepatitis C virus by virologic conditions (Grammatikos et al., 2016; Mücke et al., 2018). In patients with rheumatoid arthritis, serum Sph and Cer levels are increased (Miltenberger-Miltenyi et al., 2020), and the PC/LPC ratios of plasma represent a measure of inflammation (Fuchs et al., 2005). PC has been reported as a factor related to the effect of omalizumab in AD (Hotze et al., 2014), suggesting the association between increased serum Sph and PC (and S1P) levels and inflammation in AD.

In contrast, the significance of decreased serum Cer(16:0) levels in AD is obscure. Among the lipids whose serum levels were altered in cases of AD, the strongest change was seen for Cer(16:0) as well as for some variants of PC, suggesting that serum Cer(16:0) levels might serve as a biomarker indicating the severity of disease phenotype. However, there were no significant differences in serum Cer(16:0) levels between AD subgroups stratified by the severity of AD or between pure AD and AD with atopic comorbidities, and no significant correlation between serum Cer(16:0) levels and all clinical and laboratory data was observed. Contrary to systemic levels, Cer(16:0) increases in the skin of patients with AD (Berdyshev et al., 2018). The observed decreases in circulating Cer(16:0) levels in AD are inconsistent with reports indicating that this marker is altered (increased) in multiple other diseases such as neurodegenerative diseases (Pujol-Lereis, 2019), acute ischemic stroke (Gui et al., 2020), and chronic kidney disease (Mantovani et al., 2021). Serum levels of Cer(16:0) in patients with uncontrolled asthma are higher than those in patients with controlled asthma (Kim et al., 2020). In tissues such as the liver and adipose tissue, long-chain Cer(16:0) is deleterious that its signaling is considered therapeutic targets in some disorders, including obesity and type 2 diabetes (Choi et al., 2021; Raichur et al., 2019). Because there were significant differences in the serum levels of Sph, Cer(16:0), and S1P (Sakai et al., 2021) between patients with AD and both healthy controls and non-AD with atopic comorbidities group, we speculate that patients with AD may reflect systemic changes in sphingolipid syntheses or that the presence of large areas of eczematous skin in which the production of these lipids is altered may affect the syntheses at systemic levels; further analyses will be required to evaluate this hypothesis. Altered metabolism of sphingolipids can affect the immune system (Alaamery et al., 2021; Cartier and Hla, 2019), which might be a therapeutic target or associated with severe AD’s pathogenesis.

Because this study is observational, we cannot judge whether altered serum lipids are the pathogenic factors of AD or the results owing to systemic inflammation. Further studies may provide new insight into AD’s pathogenesis and therapeutic strategies. Indeed, circulating sphingolipids can work as predictive biomarkers, such as drug efficacy and disease prognosis in other disorders, as well as diagnostic biomarkers (Hsu et al., 2019; Xie et al., 2021). In AD, such prospective studies with metabolomics will be desired in the future. Moreover, we evaluated serum phospholipids in patients with AD at only one time point and did not evaluate skin barrier function and *FLG* mutations in this study; therefore, multiple timepoint analyses of serum phospholipids and the relationship between serum phospholipids and skin barrier function are of great interest among the future perspectives within Christine Kühne-Center for Allergy Research and Education. The experiments using animal models with altered serum sphingolipids are helpful to evaluate the pathogenesis of altered serum lipids in AD. It would be interesting to know whether such models (e.g., mice with decreased serum Cer[16:0] levels) can develop severe dermatitis or not by several stimuli. Our survey also revealed the differences in serum levels of LPE and lyso-platelet-activating factor between healthy controls and non-AD with atopic comorbidities group. In this study, although the number of patients with non-AD with atopic comorbidities was not high enough, these serum lipids might evolve to new biomarkers in allergic disorders other than AD. The association between AD and obesity has been established (Ali et al., 2018), and serum sphingolipids levels are changed depending on body mass index in patients with psoriasis (Kozłowska et al., 2019). Therefore, the evaluation of serum phospholipids in patients with AD with obesity, compared with those in patients with AD with normal body weight or healthy individuals, would be informative to investigate the mechanisms between AD and obesity. The results presented in this study provide several ideas for future approaches in this arising field.

There are some limitations to this study. First, this observational study does not provide a mechanism behind the study, and our laboratory analyses were limited to 40 serum lipids. Other Cers than Cer(16:0) were not as readily detected and therefore were excluded from the analysis. Second, allergic rhinitis, bronchial asthma, and food allergy were diagnosed solely on the basis of the participants’ self-reported diagnoses, and the number of patients without AD with atopic comorbidities was small compared with the numbers of participants in the other groups.

In summary, alterations in the serum concentration of lipids were seen in patients with AD, including increases in Sph and PC and decreases in Cer(16:0); the changes in Sph were associated with AD severity. More detailed investigations will be needed to evaluate the significance of the changes in circulating lipids in the pathogenesis of AD, especially in the context of the development of lipid-related treatments for this major chronic dermatologic condition.

## Materials and Methods

### Participants and groups

The participants were recruited in the Christine Kühne-Center for Allergy Research and Education program. They were part of an AD-focused registry program with a comprehensive collection of phenotypic and epidemiologic information as well as biobanking (Bieber et al., 2020). This study enrolled a total of 248 participants: 179 patients with AD, including 30 patients with AD only (pure AD) and 149 patients with AD with atopic comorbidities (i.e., allergic rhinitis, bronchial asthma, and/or food allergy); 22 patients without AD with atopic comorbidities; and 47 healthy controls. These study groups were the same as those used in our previous study (Sakai et al., 2021). The study was approved by the ethics committee of the University of Bonn (Bonn, Germany) and was performed in accordance with the principles of the Declaration of Helsinki. Written informed consent was obtained from each participant in the study. The diagnosis of AD was performed according to the criteria of Hanifin and Rajka (Akdis et al., 2006). Clinical scoring of AD (i.e., severity scoring of atopic dermatitis, EASI, and body surface area [%]) was performed by experienced dermatologists in the Dermatology Department at the University Hospital Bonn (Bonn, Germany) (Wollenberg et al., 2018a, 2018b). We defined AD with EASI ≥16 as moderate-to-severe AD and AD with EASI <16 as mild-to-moderate AD, which is currently the cut-off for the indication for systemic therapy (Beck et al., 2014). Information on pruritus and sleeplessness was extracted from the subjective severity scoring of atopic dermatitis. Diagnosis of allergic rhinitis, bronchial asthma, or food allergy was conducted on the basis of the information provided by the subjects on a self-reported diagnosis. Blood samples were collected from participants and immediately processed to serum using a standardized procedure; the resulting sera were stored frozen at –80 ºC until assessment. The patients' demographics are shown in the previous literature (Sakai et al., 2021). Briefly, participants were limited to adults (aged 20–59 years), and patients with any systemic or chronic disorder requiring hospital visits other than AD and atopic comorbidities (allergic rhinitis, bronchial asthma, or food allergy) were excluded. There were no differences among the three study groups with regard to age, body mass index, or sex. There was no apparent bias in the AD group with regard to age, body mass index, EASI, body surface area, total IgE levels, or AD duration. Moreover, in the AD group, onsets of AD of the patients were 1.0 (0.17–8.25) years (median [Q1–Q3]), and 82.6% of the patients developed AD in childhood and adolescence (less than 18 years). None of the patients with AD had received systemic treatment for AD, including systemic corticosteroids and biologics, within 30 days before sample collection. In contrast, 82.4% of the patients with AD had received the treatment with topical corticosteroids within the preceding year. Regarding ethnicity, 11 participants were Asian, and all other participants were German. Serum levels of thymus and activation-regulated chemokine were measured by MSD U-PLEX Multiplex Assays (Meso Scale Diagnostics, Rockville, MD) according to the manufacturer’s instructions. This biomarker was measured in all patients with AD.

### Lipid analyses

Serum lipid analyses were performed in all participants using liquid chromatography coupled to tandem mass spectrometry. We evaluated 40 lipids in the serum of all participants. These lipids belong to the most prevalent group of phospholipids and were chosen because these lipids were readily detected and could be well-quantified. Cer(16:0) is the most abundant Cer that was assessed. As mentioned in the limitations of the main text, other Cers than Cer(16:0) were not as readily detected and therefore were excluded from the analysis. Protein precipitation was performed in 96-well plates from Sarstedt (Nuremberg, Germany; K-series) from 20.0 μl serum samples by addition of 200 μl methanol (99.9%, Chromasolv LC-MS grade, Honeywell Riedel-de Haën, Seelze, Germany) supplemented with 10.0 μl of an internal standard mixture (45 pmol each per injection volume, see the following paragraph) as an internal reference for normalization and quantification. After vigorous mixing and precipitation overnight at –80 °C, supernatants were transferred to a fresh plate after centrifugation for 10 minutes at 2,000*g* at 4 °C. After evaporating the solvent, pellets were reconstituted in 200.0 μl DMSO/methanol (1:1, v/v) and stored at –80 °C until further analysis. The internal standard mixture was prepared in methanol as follows: C15‒Cer, C17‒Sph, C17‒LPC, C17‒PC, C17‒sphingomyelin, and C17‒LPE. All compounds were purchased from Avanti Polar Lipids (Alabaster, AL) or Sigma-Aldrich (Deisenhofen, Germany). The HPLC system consisted of an autosampler AS-100 (Biorad, München, Germany), a G1322A degassing unit, a G1212A binary pump (Series 1100), and an L-2300 column oven (Merck Hitachi, Tokyo, Japan) with a 60 × 2 mm MultoHigh 100 RP 18 column with 3 μm particle size (CS-Chromatographie Service, Langerwehe, Germany), which was maintained at 50 °C. Mobile phase A consisted of 1.0% (v/v) formic acid in double distilled water, and mobile phase B consisted of 100% methanol. The column was equilibrated in 10% B with a flow rate of 0.5 ml/min. The mobile phase switched to 100% B after sample injection. The flow rate increased linearly from 0.5 ml/min at 5 minutes to 1.0 ml/min at 7 minutes and remained constant until 10 minutes. Subsequently, the mobile phase changed to 10% B, and the flow rate decreased linearly from 1.0 ml/min at 10 minutes to 0.5 ml/min at 10.5 minutes and remained constant until the end of the program at 11.3 minutes. Detection took place between 2 and 10 minutes. The injection volume per sample was 30.0 μl; samples were cooled at 4 °C. The HPLC system was coupled to the API 2000 triple-quadrupole mass spectrometer (Sciex, Foster City, CA) equipped either with an ESI or an APCI source, both operating in a positive mode under the following source parameters: source temperature of 450 °C, curtain gas of 40, low collision gas, ion spray voltage of 5,500 (ESI), nebulizer current of 4 (APCI), ion source gas1 of 30 (ESI) or 60 (APCI), and ion source gas2 of 60 (ESI) or 30 (APCI). Target ions Q1 > Q3, assignment to an internal standard, and mode of analysis are given in Table 5. The analytical results were quantified with Analyst 1.6.2 (AB Sciex, Forster City, CA) on the basis of internal standard samples and an external standard curve.

**Table 5:** List of Target Ions/MRM Transitions, Assignment to Internal Standard, and Mode of Analysis

Name	Internal Standard	Mode	Target Ion Q1>Q3
C15:0 Cer		APCI	524.4>264.3
C16:0 Cer	>C15:0 Cer<	APCI	538.7>264.4
d17:1 Sph		ESI	289.4>268.3
d18:1 Sph	>d17:1 Sph<	ESI	300.5>282.3
C17:0 SM (18:0)		ESI	717.483>184.1
C12:0 SM (18:1)	>C17:0 SM (18:0)<	ESI	647.7>184.1
C14:0 SM (18:1)	>C17:0 SM (18:0)<	ESI	675.7>184.1
C16:0 SM (18:1)	>C17:0 SM (18:0)<	ESI	703.8>184.1
C18:1 SM (18:1)	>C17:0 SM (18:0)<	ESI	729.8>184.1
C18:0 SM (18:1)	>C17:0 SM (18:0)<	ESI	731.8>184.1
C20:0 SM (18:1)	>C17:0 SM (18:0)<	ESI	759.9>184.1
C22:0 SM (18:1)	>C17:0 SM (18:0)<	ESI	797.9>184.1
C24:1 SM (18:1)	>C17:0 SM (18:0)<	ESI	813.9>184.1
C24:0 SM (18:1)	>C17:0 SM (18:0)<	ESI	815.9>184.1
28:0 PC	>34:0 PC<	ESI	678.5>184.1
30:0 PC	>34:0 PC<	ESI	706.55>184.1
30:1 PC	>34:0 PC<	ESI	704.5>184.1
32:0 PC	>34:0 PC<	ESI	734.55>184.1
32:1 PC	>34:0 PC<	ESI	732.55>184.1
34:0 PC		ESI	762.455>184.1
34:1 PC	>34:0 PC<	ESI	760.6>184.1
34:2 PC	>34:0 PC<	ESI	758.629>184.1
36:0 PC	>34:0 PC<	ESI	790.65>184.1
36:1 PC	>34:0 PC<	ESI	788.6>184.1
36:2 PC	>34:0 PC<	ESI	786.6>184.1
36:3 PC	>34:0 PC<	ESI	784.6>184.1
36:4 PC	>34:0 PC<	ESI	782.55>184.1
38:1 PC	>34:0 PC<	ESI	816.65>184.1
38:2 PC	>34:0 PC<	ESI	814.65>184.1
38:3 PC	>34:0 PC<	ESI	812.6>184.1
38:4 PC	>34:0 PC<	ESI	810.6>184.1
16:0 LPC	>17:0 LPC<	ESI	496.278>184.1
16:1 LPC	>17:0 LPC<	ESI	494.3>184.1
17:0 LPC		ESI	510.222>184.1
18:0 LPC	>17:0 LPC<	ESI	524.35>184.1
18:1 LPC	>17:0 LPC<	ESI	522.35>184.1
18:2 LPC	>17:0 LPC<	ESI	520.35>184.1
20:1 LPC	>17:0 LPC<	ESI	550.4>184.1
20:3 LPC	>17:0 LPC<	ESI	546.35>184.1
20:4 LPC	>17:0 LPC<	ESI	544.35>184.1
16:0 LPE	>17:1 LPE<	ESI	454.3>313.25
17:1 LPE		ESI	466.3>325.3
18:0 LPE	>17:1 LPE<	ESI	482.3>341.3
18:1 LPE	>17:1 LPE<	ESI	480.3>339.3
18:2 LPE	>17:1 LPE<	ESI	478.3>337.25
20:4 LPE	>17:1 LPE<	ESI	502.3>361.25
LysoPAF	>17:0 LPC<	ESI	482.3>104.20

Abbreviations: APCI, atmospheric pressure chemical ionization; Cer, ceramide; ESI, electrospray ionization; SM, sphingomyeline; Sph, sphingosine; LPC, lysophosphatidylcholine; LPE, lysophosphatidylethanolamine; PC, phosphatidylcholine.

For Cer, SM, and lyso-compounds, the number of carbon atoms is given and also the number of unsaturated bonds; for SM, the sphingoid backbone is given in brackets; and for PC, the total number of carbon atoms as well as of unsaturated bonds are given.

### Statistical analyses

Data are presented using the median with 25th and 75th percentiles (Q1–Q3) or the box plots. We applied the two-tailed Mann‒Whitney U test when comparing two groups and the Kruskal‒Wallis test followed by pairwise comparisons with Mann‒Whitney U test when comparing three groups. Spearman’s correlation was used to compare continuous variables. The *P*-values for multiple comparisons were adjusted using Benjamini‒Hochberg procedure. A *P* < 0.05 was considered statistically significant. In addition, we sought the serum phospholipids being associated with the risk of AD using L1 penalized logistic regression between AD and non-AD, which includes healthy controls and non-AD with atopic comorbidities. The regularization parameter lambda was chosen by 10-fold cross-validation on the basis of the binomial deviance criterion. All analyses were performed using SPSS Statistics, version 25 (IBM, Armonk, NY); Prism, version 9.2.0 (GraphPad Software, San Diego, CA); and R, version 4.1.0 (R Core Team (2021) (R: A language and environment for statistical computing, Foundation for Statistical Computing, Vienna, Austria; https://www.R-project.org/) with packages glmnet_4.1-2, car_3.0-10, and openxlsx_4.2.4.

### Data availability statement

Datasets related to this article can be found at https://data.mendeley.com/datasets/tgjfsdb4t4/1, hosted at Mendeley Data.

## ORCIDs

Takashi Sakai: http://orcid.org/0000-0001-7128-3237

Nadine Herrmann: http://orcid.org/0000-0003-4924-2281

Laura Maintz: http://orcid.org/0000-0001-6053-1530

Tim Joachim Nümm: http://orcid.org/0000-0001-6110-6712

Thomas Welchowski: http://orcid.org/0000-0003-2940-647X

Ralf A. Claus: https://orcid.org/0000-0001-7232-4088

Markus H. Gräler: http://orcid.org/0000-0001-6650-7849

Thomas Bieber: http://orcid.org/0000-0002-8800-3817

## Author Contributions

Conceptualization: TS, NH, TB; Data Curation: TS, NH, LM, TJN, TW, RAC, MHG, TB; Formal Analysis: TS, NH, LM, TJN, TW, RAC, MHG; Funding Acquisition: TB; Investigation: TS, NH, LM, TJN, TW, RAC, MHG; Project Administration: TS, NH, TB; Supervision: TS, NH, TB; Visualization: TS; Writing - Original Draft Preparation: TS; Writing - Review and Editing: TS, NH, LM, TJN, TW, RAC, MHG, TB
